# Curcumin Targets Both Apoptosis and Necroptosis in Acidity-Tolerant Prostate Carcinoma Cells

**DOI:** 10.1155/2021/8859181

**Published:** 2021-05-22

**Authors:** Yoon-Jin Lee, Kwan-Sik Park, Sang-Han Lee

**Affiliations:** Department of Biochemistry, College of Medicine, Soonchunhyang University, Cheonan, Republic of Korea

## Abstract

**Objective:**

Curcumin, a major bioactive curcuminoid derived from the rhizome of *Curcuma longa*, is known to have anticancer potential and is still under investigation. In this study, we investigated the cytotoxic mechanism(s) of curcumin against acidity-tolerant prostate cancer PC-3AcT cells in lactic acid-containing medium.

**Methods:**

Using 2D-monolyer and 3D spheroid culture models, MTT assay, annexin V-PE binding assay, flow cytometric analysis, measurement of ATP content, and Western blot analysis were used for this study.

**Results:**

At nontoxic concentrations in normal prostate epithelial RWPE-1 and HPrEC cells, curcumin led to strong cytotoxicity in PC-3AcT cells, including increases in sub-G_0_/G_1_ peak, annexin V-PE-positive cells, and ROS levels; loss of mitochondrial membrane potential; reduction of cellular ATP content; DNA damage; and concurrent induction of apoptosis and necroptosis. A series of changes induced by curcumin were effectively reversed by reducing ROS levels or replenishing ATP. Pretreatment with apoptosis inhibitor Q-VD-Oph-1 or necroptosis inhibitor necrostatin-1 restored cell viability inhibited by curcumin. Treatment of 3D spheroids with curcumin decreased cell viability, accompanied by an increase in mediators of apoptosis and necroptosis, including cleaved caspase-3 and cleaved PARP, phospho (p)-RIP3, and p-MLKL proteins.

**Conclusion:**

This study shows that curcumin simultaneously induces apoptosis and necroptosis by oxidative mitochondrial dysfunction and subsequent ATP depletion, providing a mechanistic basis for understanding the novel role of curcumin for prostate carcinoma cells.

## 1. Introduction

Prostate cancer (PC) constitutes 20% of all male cancers and is the main cause of cancer-related male mortality in the United States [1]. Although androgen deprivation therapy still remains an important strategy for patients diagnosed with locally advanced PC, the majority of patients eventually advance to androgen-independent stage and exhibit high resistance to anticancer therapy.

Hypoxia due to insufficient blood perfusion and enhanced aerobic glycolysis in tumor cells leads to increased extracellular excretion of lactic acid and subsequent acidification (pH 6.5-6.9) of the tumor microenvironment [2]. Tumor acidity plays an important role in the tumor cell aggressiveness and determines the efficacy of therapeutic agents that target tumors. Substantial evidence suggests that in an acidic environment, tumors show poor prognosis and a high incidence of metastasis [3], increased mutation rate [4], and resistance to chemotherapy [5] and radiotherapy [6]. Therefore, treatment strategies that take into account the effects of extracellular acidic pH on cancer metabolism will facilitate the discovery of more effective therapeutics that inhibit tumor energetics and viability in the tumor microenvironment.

Curcumin [1,7-bis-(4-hydroxy-3-methoxyphenyl) hepta-1,6-diene-3,5-dione], a polyphenol derived from the rhizome of Curcuma longa, exhibits anticancer activity by inhibiting tumor initiation and progression via the regulation of several signaling pathways and inhibition of cell proliferation, invasion, and angiogenesis [7, 8]. Curcumin restricts cell proliferation and induces apoptotic cell death in various cancer cells, where epigenetic effects of curcumin can also contribute to anticancer activities [9]. Curcumin reduces the expression of androgen receptors in PC [8]. It induces apoptotic cell death by targeting various signaling pathways, such as NF-*κ*B and Wnt/*β*-catenin pathways [10], PI3K/Akt pathway [8], and MAOA/mTOR/HIF-1 signaling pathway [11].

Programmed cell death (PCD) plays a critical role in eradicating potentially harmful mutated cells that can lead to cell transformation and tumor formation. Apoptosis is a well-known type of PCD, which is highly regulated. By contrast, therapeutic approaches targeting other forms of nonapoptotic PCD, including necroptosis, ferroptosis, pyroptosis, parthanatos, and NETosis/ETosis, may have great potential in chemotherapy, especially in apoptosis-resistant tumors [12]. Several reports have suggested that PC cells acquire the therapeutic resistance through apoptosis evasion [13] or protective autophagy [14]. Therefore, developing therapeutic approaches that trigger nonapoptotic cell death or inhibit autophagy in cancer cells can overcome the treatment resistance and facilitate the effective treatment of prostate cancer. Necroptosis, a subform of regulated necrosis, is initiated by auto- and transphosphorylation of receptor-interacting protein kinase 1 (RIP1) and RIP3 to form the necrosome. Necrosomes subsequently activate mixed lineage kinase domain-like protein (MLKL) via phosphorylation, and its translocation across the plasma membranes leads to necroptosis [15]. Dysregulated apoptotic signaling in cancer cells triggers chemotherapeutic resistance, and therefore, concurrent induction of apoptosis and necroptosis is an attractive treatment strategy to overcome drug resistance during cancer treatment.

The objective of the current study is to identify the cytotoxic mechanism(s) of curcumin against PC cells under lactic acidosis. To this end, we established an acidic pH-tolerant PC cell line PC-3AcT through prolonged preconditioning of parental PC-3 cells with lactic acid and performed mechanistic studies under acidic condition in 2D monolayer cell culture, as well as in 3D spheroid culture, as *in vitro* model systems mimicking the acidic tumor microenvironment.

## 2. Methods

### 2.1. Cell Culture

The human PC cell line PC-3 and two human prostate epithelial cell lines, RWPE-1 and HPrEC, were obtained from the American Type Culture Collection (ATCC). The RWPE-1 cells were grown in keratinocyte serum-free medium supplemented with bovine pituitary extract (0.05 mg/mL), human recombinant epidermal growth factor (5 ng/mL), and antibiotic-antimycotic (Gibco). The HPrEC cells were grown in prostate epithelial cell basal medium supplemented with prostate epithelial cell growth kit (ATCC). Acidic pH-tolerant PC-3AcT cells were established by continuous exposure of PC-3 cells to lactic acid (3.8 *μ*M) via serial passage over 4 times over 15 days. The PC-3 and PC-3AcT cells were grown in Dulbecco's Modified Eagle's medium (DMEM) containing 3.8 *μ*M lactic acid and 5% fetal bovine serum. The average doubling times of PC-3 and PC-3AcT cells were determined to be 23 h and 21 h, respectively. For further analysis, cells were treated with dimethylsulfoxide (Sigma-Aldrich) as a negative control, docetaxel (Sigma-Aldrich) as a positive control, and curcumin (Sigma-Aldrich) for 48 h, where the treatment concentrations of docetaxel and curcumin were selected by referring to experimental conditions in other studies using prostate cancer cells [16, 17].

### 2.2. Cell Viability Assay

We followed the methods of Lee et al. [18]. Briefly, cells were seeded on 96-well microtiter plates at 5 × 10^3^ cells/well, 24 h prior to treatment with various concentrations of docetaxel (0, 20, 40, 60, 80, 100, 120, 140, and 160 nM) and curcumin (0, 20, 40, 60, 80, 100, 120, 140, and 160 *μ*M), dissolved in the lactic acid-containing DMEM. After incubation, 3-(4,5-dimethylthiazol-2-yl)-2,5-diphenyltetrazolium bromide (Sigma-Aldrich) was added to culture medium and incubated for 4 h at 37°C. Absorbance values were measured at 540 nm by GloMax-Multi microplate multimode reader (Promega Corporation). The percentage viability of cells was determined by comparison with the results obtained using vehicle-treated control cells (100%).

### 2.3. Annexin V-PE Binding Assay

Analysis of apoptosis and necrotic cell distribution was performed using the Muse Annexin V & Dead Cell Assay kit (Merck KGaA) according to the method of Lee et al. [18]. Briefly, cells were treated with curcumin in the lactic acid-containing DMEM at 37°C for 48 h, after which the cells were trypsinized, collected into a culture medium supplemented with the Muse Annexin V & Dead Cell reagent, and analyzed by a Muse cell analyzer (Merck KGaA). Annexin V-phycoerythrin (PE) and 7-amino-actinomycin D (7-AAD) double staining was performed to quantify annexin V-PE-positive apoptotic cells and 7-AAD-positive necrotic cells.

### 2.4. Cell Cycle Analysis

The percentages of cells at each phase of the cell cycle were determined by staining with propidium iodide (PI) according to the method of Lee et al. [18]. Briefly, trypsinized cells were centrifuged at 500 × g at 4°C for 7 min and then fixed with 70% ethanol overnight at -20°C. After washing with 1× phosphate-buffered saline (PBS), the cells were incubated with the Muse cell cycle reagent (Merck Millipore). Data from 10,000 cells were analyzed using the MACSQuant analyzer and MACSQuantify software version 2.5 (Miltenyi Biotec GmbH).

### 2.5. Western Blot Analysis

We followed the methods of Lee et al. [18]. Briefly, total cell lysates were extracted with 1× RIPA buffer and the protein concentrations were measured using the BCA protein assay kit (Pierce). Cell lysates containing 40 *μ*g of protein were separated on 4-12% NuPAGE gel (Thermo Fisher Scientific, Inc.) and transferred to a polyvinylidene fluoride membrane (GE Healthcare Life Sciences). The membrane was incubated with primary and secondary antibodies coupled to horseradish peroxidase (HRP) for protein detection. Reactive proteins were visualized using an enhanced chemiluminescence (Cyanagen Srl) detection kit and X-ray film. The membrane was reprobed with anti-*β*-actin (Sigma-Aldrich; 1 : 1,000 dilution), anti-RIP3, and anti-MLKL antibodies that served as the loading controls. Antibodies to p-ATM(Ser^1981^) (Catalog no. 5883; 1 : 500 dilution), p-ATR(Ser^428^) (Catalog no. 2853; 1 : 500 dilution), p-CHK1(Ser^345^) (Catalog no. 2348; 1 : 500 dilution), p-CHK2(Thr^68^) (Catalog no. 2197; 1 : 500 dilution), p-histone H2A.X(Ser^139^) (Catalog no. 9718; 1 : 500 dilution), MLKL (Catalog no. 14993; 1 : 1,000 dilution), p-MLKL (Catalog no. 91689; 1 : 500 dilution), RIP3 (Catalog no. 13526; 1 : 1,000 dilution), p-RIP3 (Catalog no. 93654; 1 : 500 dilution), Bax (Catalog no. 5023; 1 : 500 dilution), Bcl-2 (Catalog no. 2820; 1 : 500 dilution), PARP (Catalog no. 9542; 1 : 500 dilution), cleaved PARP (Catalog no. 9541; 1 : 500 dilution), caspase-3 (Catalog no. 9665; 1 : 500 dilution), and cleaved caspase-3 (Catalog no. 9664; 1 : 500 dilution) were purchased from Cell Signaling Technology, Inc. (Danvers, CO, USA) and used for antigen detection. Goat anti-rabbit IgG-HRP (Catalog no. sc-2004; 1 : 5,000 dilution) and goat anti-mouse IgG-HRP (Catalog no. sc-2005; 1 : 5,000 dilution) were purchased from Santa Cruz Biotechnology, Inc.

### 2.6. Measurement of ROS and Mitochondrial Membrane Potential

We followed the methods of Lee et al. [18]. Briefly, cells were seeded on 6-well culture plates at 10^5^ cells/well, 24 h prior to treatment with docetaxel (0, 10, 20, and 40 nM) or curcumin (0, 10, 20, and 40 *μ*M) in lactic acid-containing DMEM for 48 h. Following trypsinization, cells were harvested by centrifugation at 500 × g for 7 min and then resuspended in serum-free DMEM containing 10 *μ*M 2′,7′-dichlorodihydrofluorescein diacetate (DCF-DA) (Sigma-Aldrich) and 30 nM Rhodamine 123 (Sigma-Aldrich) to measure the levels of ROS and *ΔΨ*m, respectively, in the dark at 37°C for 30 min. After washing cells twice with 1× PBS, the average fluorescence intensity of 10,000 cells was measured using the MACSQuant analyzer and MACSQuantify software version 2.5 (Miltenyi Biotec GmbH).

### 2.7. Measurement of ATP Content

Cellular ATP levels were measured using the CellTiter-Glo Luminescent Cell Viability Assay kit (Promega Corporation) according to the method of Seo et al. [16]. Briefly, cells were treated with curcumin for 48 h in the lactic acid-containing DMEM, after which the CellTiter-Glo reagent (100 *μ*L/well) was added to the cell culture, placed in a shaker for 2 min, and incubated at room temperature (RT) for 10 min, to induce complete lysis. Luminescence values were measured using the GloMax-Multi microplate multimode reader (Promega Corporation). The data was normalized to cell number, after counting the number of viable cells by trypan blue exclusion assay for each treatment and time point.

### 2.8. Spheroid Culture and Viability Assay

We followed the methods of Lee et al. [18]. Briefly, cells were seeded in an ultralow attachment 96-well plates at 10^4^ cells/mL in 100 *μ*L medium/well. The plates were centrifuged at 1,000 rpm for 10 min to facilitate clustering of the cells into the wells, as described by Chambers et al. [19], and maintained in the complete DMEM containing 3.8 *μ*M of lactic acid. Spheroids were treated with curcumin for 48 h. Phase-contrast images were taken using a Leica inverted microscope. Spheroid viability was determined using the enhanced cell viability assay kit (CellVia). Briefly, 10 *μ*L of CellVia solution was added to each well, kept at RT for 1 h, and then mixed by shaking for 1 min. Formazan formed in living cells was measured at 450 nm using a GloMax-Multi microplate multimode reader (Promega Corporation).

### 2.9. Spheroid Staining

We followed the methods of Lee et al. [18]. Briefly, cells were incubated with fluorescein diacetate (FDA, 5 *μ*g/mL) (Sigma-Aldrich) and PI (10 *μ*g/mL) (Sigma-Aldrich) in the dark, to stain live and dead cells, respectively. The FDA, a cell-permeable esterase substrate, serves as an indicator for viable cells by assessing enzymatic activity and cell-membrane integrity, while PI passes through damaged areas of dead or dying cell membranes into the nucleus and binds to DNA. Cells were imaged using a Leica EL6000 fluorescence microscope and LAS version 4.3 software (Leica Microsystems GmbH).

### 2.10. Statistical Analysis

Statistical comparisons among multiple groups were analyzed by one-way ANOVA and Tukey's *post hoc* correction, using the SPSS version 17.0 software package (SPSS, Inc.). Data are expressed as mean ± standard deviation (S.D.) for three independent experiments. *P* < 0.05 was considered statistically significant.

## 3. Results

### 3.1. DNA Damage, Apoptosis, and Necroptosis in Curcumin-Induced Cytotoxicity

**Figure 1:**
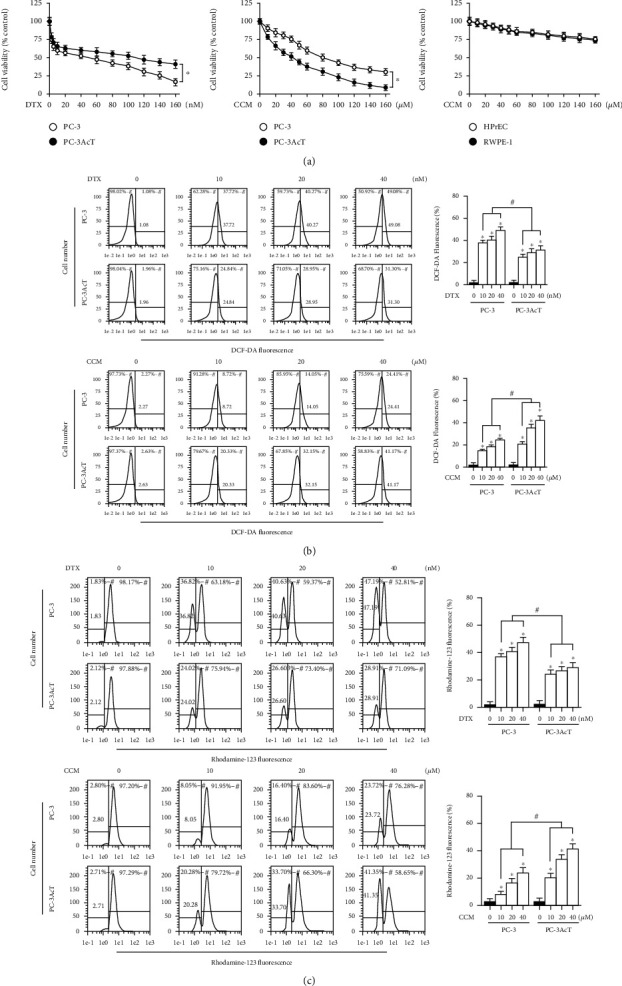
Effects of docetaxel and curcumin on cell viability, ROS level, and mitochondrial membrane potential. PC-3 and PC-3AcT cells were treated with the indicated concentrations of DTX or CCM in DMEM containing 3.8 *μ*M lactic acid for 48 h. Two human prostate epithelial cell lines (RWPE-1 and HPrEC) were incubated in lactic acid-free DMEM. (a) Cell viability was measured by MTT assay. (b) Cellular ROS levels were measured by staining cells with DCF-DA (10 *μ*M). (c) *ΔΨ*m was measured by staining cells with Rhodamine 123 (30 nM). ^∗^*P* < 0.05 vs. respective PC-3 cells. DTX: docetaxel; CCM: curcumin; *ΔΨ*m: mitochondrial membrane potential.

**Figure 2:**
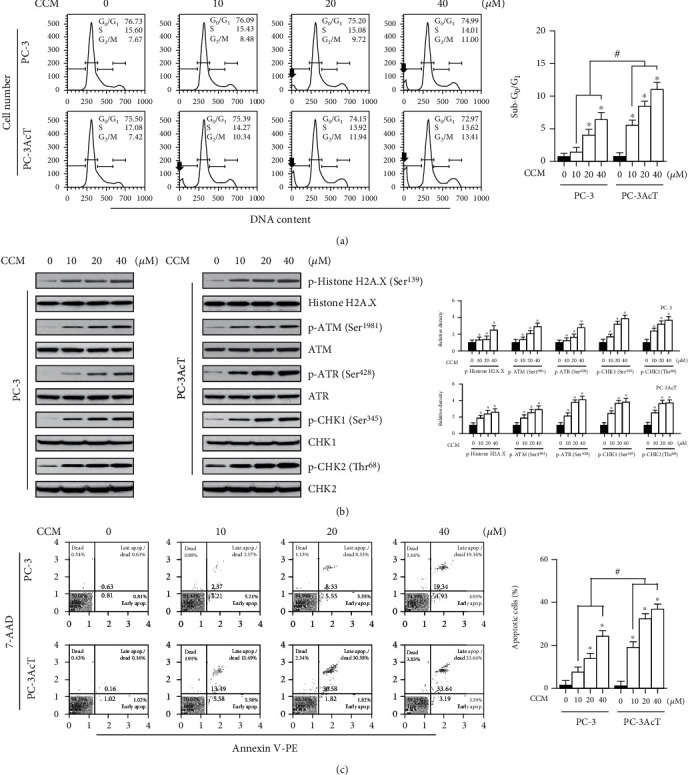
Effects of curcumin on cell cycle, DNA damage response, and cell death. PC-3 and PC-3AcT cells were treated with the indicated concentrations of CCM in DMEM containing lactic acid (3.8 *μ*M) for 48 h. (a) Cell cycle distribution was determined by flow cytometry following PI (20 *μ*g/mL) staining. (b) The levels of DNA damage response proteins were assessed by Western blotting. (c) Cell death fraction was analyzed using annexin V-PE binding assay. ^∗^*P* < 0.05 vs. respective control cells. ^#^*P* < 0.05 vs. respective PC-3 cells. CCM: curcumin. Arrows, sub-G_0_/G_1_ peak.

To further investigate the novel effects of curcumin on PC cells, this study was carried out in lactic acid-containing media. In MTT assay, treatment of cells with increasing docetaxel and curcumin concentrations over 48 h decreased cell viability. PC-3AcT cells (IC_50_ = 109.57 nM) showed to be more tolerant to docetaxel compared to parental PC-3 cells (IC_50_ = 49.83 nM), whereas increased sensitivity to curcumin was compared with PC-3 cells (IC50 = 80.51 *μ*M) and more evident in PC-3AcT cells (IC_50_ = 42.19 *μ*M) (Figure 1(a)). Cell viability above 90% was observed in two human prostate epithelial cell lines RWPE-1 and HPrEC treated with ≤40 *μ*M curcumin, and further experiments were conducted at these concentrations. Next, the effects of docetaxel and curcumin on ROS levels and mitochondrial membrane potential (*ΔΨ*m) were measured.  Figure 1(b) shows that ROS level and *ΔΨ*m loss increased, depending on the concentrations of docetaxel and curcumin. Compared to the untreated control group, the proportion of cells with elevated ROS levels of the docetaxel-treated group was increased to 49.08% and 31.30% in PC-3 and PC-3AcT cells, respectively, whereas in the curcumin-treated group, it increased to 24.41% and 41.17%, respectively. The degree of *ΔΨ*m loss in response to docetaxel and curcumin was similar to the change in ROS levels. The proportion of cells with *ΔΨ*m loss of the docetaxel-treated group was increased to 47.19% and 28.91% in PC-3 and PC-3AcT cells, respectively, whereas in the curcumin-treated group, it increased to 23.72% and 41.35%, respectively (Figure 1(c)). Cell cycle analysis after curcumin treatment showed a time-dependent increase in a sub-G_0_/G_1_ peak, suggestive of apoptosis, and delayed G_2_/M transition in the cell cycle (Figure 2(a)). However, except for the difference in sub-G_0_/G_1_ level, no significant difference in cell cycle distribution was observed between PC-3 cells and PC-3AcT cells. To evaluate the effects of curcumin on DNA damage, the phosphorylation levels of markers for DNA damage response and DNA double-strand break were examined after treatment with curcumin.  Figure 2(b) shows that curcumin induced increased levels of p-ATM(Ser^1981^) and p-ATR(Ser^428^), their respective downstream substrates p-CHK1(Ser^345^) and p-CHK2(Thr^68^), and DNA double-strand break marker, p-histone H2A.X(Ser^139^). The percentage of annexin V-PE-positive cells undergoing apoptosis in early and late phases increased to 36.83% in PC-3AcT cells treated with 40 *μ*M curcumin, compared to 24.27% in PC-3 cells (Figure 2(c)).

### 3.2. Curcumin Induces Both Apoptosis and Necroptosis

**Figure 3:**
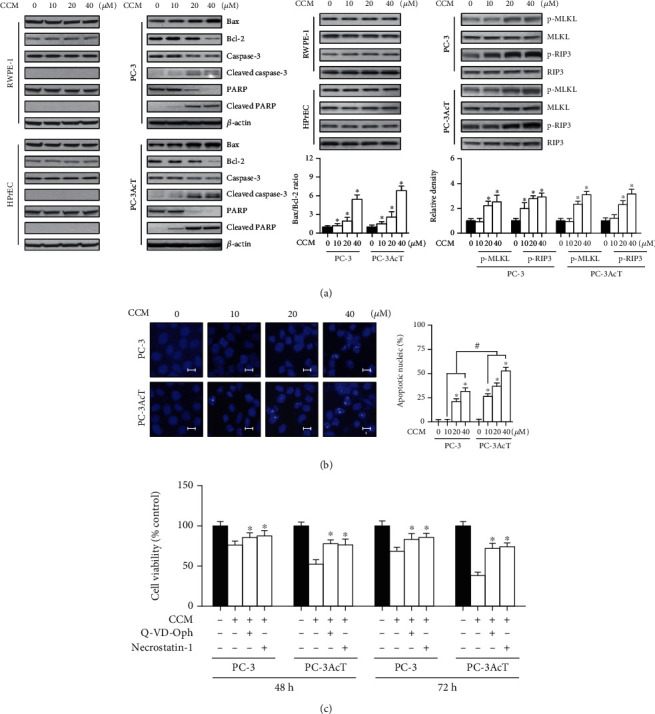
Apoptosis- and necroptosis-inducing effects of curcumin. PC-3 and PC-3AcT cells were treated with the indicated concentrations of CCM for 48 h. Two human prostate epithelial cell lines (RWPE-1 and HPrEC) were incubated in lactic acid-free DMEM. (a) The levels of apoptosis- and necroptosis-mediated proteins were assessed by Western blotting. (b) Nuclear morphology was assessed by staining with DAPI (scale bar = 5 *μ*m). (c) Cells were pretreated with necrostatin-1 (25 *μ*M) and Q-VD-Oph-1 (10 *μ*M) 2 h prior to treatment with CCM (40 *μ*M) for 48 h in DMEM containing lactic acid (3.8 *μ*M). Cell viability was measured by MTT assay. ^∗^*P* < 0.05 vs. respective control cells. ^#^*P* < 0.05 vs. respective PC-3 cells. CCM: curcumin.

To investigate the type of cell death in the cytotoxicity of curcumin, the expression of apoptosis- and necroptosis-related proteins was examined by Western blotting.  Figure 3(a) shows that curcumin increased the levels of proapoptotic proteins, such as Bax and cleaved forms of caspase-3 and PARP, and decreased the level of antiapoptotic protein Bcl-2. Curcumin also increased the phosphorylation of RIP3 and its downstream target MLKL in a concentration-dependent manner. However, following curcumin treatment of RWPE-1 and HPrEC cells, no significant changes were noticed in the levels of these proteins. Upon staining of nuclei with DAPI, the proportion of adherent cells with condensed and fragmented nuclei was much higher in PC-3AcT cells than in PC-3 cells (Figure 3(b)). To further confirm the results, cells were treated with Q-VD-Oph-1 (a pancaspase inhibitor for apoptosis) or necrostatin-1 (a RIP1 inhibitor for necroptosis) prior to curcumin treatment for 48 h.  Figure 3(c) shows that pretreatment with inhibitors effectively restored cell viability, compared with curcumin alone.

### 3.3. Cellular ATP and ROS as Major Determinants of Curcumin-Induced Cell Death

**Figure 4:**
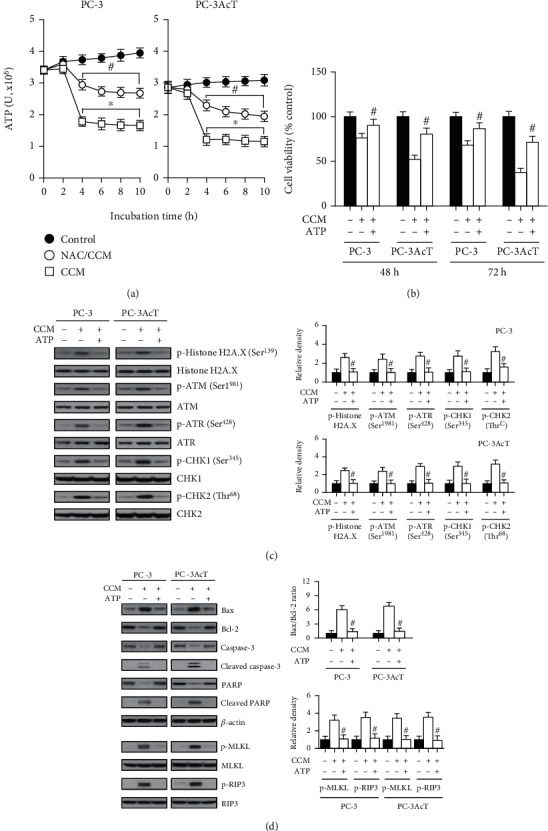
Effects of ATP supplementation on curcumin-treated PC-3 and PC-3AcT cells. (a) Cells were pretreated with or without NAC (5 mM) for 2 h prior to treatment with CCM (40 *μ*M) for the indicated times in DMEM containing lactic acid (3.8 *μ*M). Cellular ATP levels were measured by CellTiter-Glo luminescent cell viability assay. Cells were pretreated with ATP (1 mM), 2 h prior to treatment with CCM (40 *μ*M) for 48 h in DMEM containing lactic acid (3.8 *μ*M). Cell viability was measured by MTT assay (b). The levels of DNA damage response- (c) apoptosis-, and necroptosis- (d) mediated proteins were assessed by Western blotting. ^∗^*P* < 0.05 vs. respective control cells. ^#^*P* < 0.05 vs. respective CCM-treated cells. CCM: curcumin; NAC: N-acetylcysteine.

We then measured changes in intracellular ATP levels according to the curcumin-induced mitochondrial dysfunction. In parallel with impaired mitochondrial function, cellular ATP levels began to decrease within 2 h after curcumin treatment and rapidly decreased until 4 h (Figure 4(a)). Supplementation of ATP (final concentration: 1 mM) prevented changes caused by curcumin on cell viability (Figure 4(b)) and levels of DNA damage response markers, including p-ATM(Ser^1981^), p-ATR(Ser^428^), p-CHK1(Ser^345^), p-CHK2(Thr^68^), and p-histone H2A.X(Ser^139^) (Figure 4(c)), and cell death markers including p-MLKL and p-RIP3, cleaved caspase-3, cleaved PARP, and Bax/Bcl-2 ratio (Figure 4(d)).

**Figure 5:**
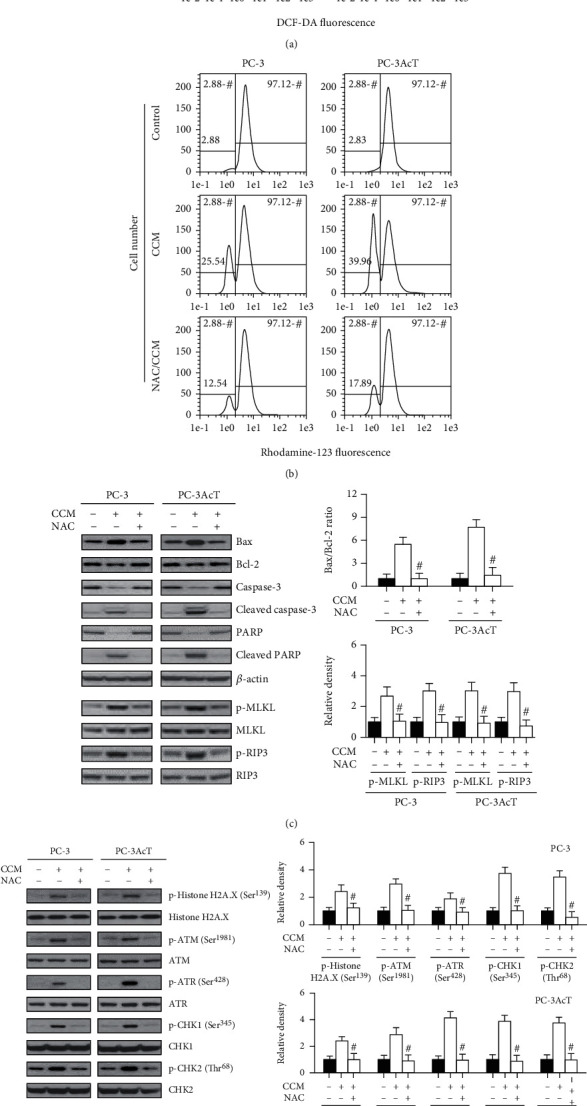
Effects of N-acetylcysteine pretreatment on curcumin-treated PC-3 and PC-3AcT cells. Cells were pretreated with or without NAC (5 mM) for 2 h, followed by CCM treatment at the indicated concentration or 40 *μ*M, if not indicated, for 48 h in DMEM containing lactic acid (3.8 *μ*M). (a) Cellular ROS levels were measured by staining cells with DCF-DA (10 *μ*M). (b) *ΔΨ*m was measured by staining cells with Rhodamine 123 (30 nM). The levels of apoptosis-, necroptosis- (c), and DNA damage response- (d) mediated proteins were measured by Western blotting. (e) Cell viability was measured by MTT assay. ^#^*P* < 0.05 vs. respective CCM-treated cells. CCM: curcumin; NAC: N-acetylcysteine.

To determine whether high levels of ROS are associated with increased cytotoxicity caused by curcumin, the effect of ROS scavenging on the cytotoxicity of curcumin was investigated. Pretreatment of PC-3 and PC-3AcT cells with N-acetylcysteine (NAC) effectively improved a series of changes induced by curcumin on cellular ROS levels (Figure 5(a)), percentage of cells showing *ΔΨ*m loss (Figure 5(b)), levels of molecular markers for apoptosis and necroptosis (Figure 5(c)), DNA damage response (Figure 5(d)), and cell viability (Figure 5(e)).

**Figure 6:**
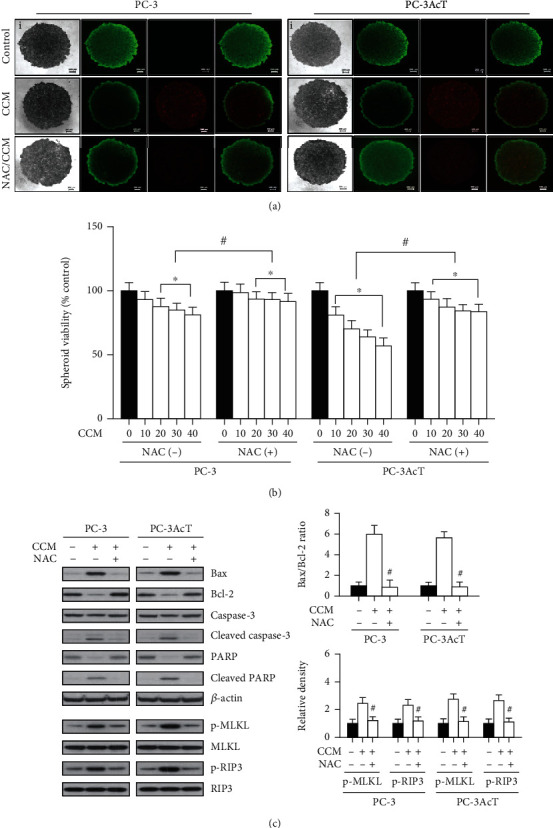
Effects of pretreatment with N-acetylcysteine on curcumin-induced cytotoxicity in 3D cultures of PC-3 and PC-3AcT cells. Spheroids were cultured in ultralow cluster 96-well plate and pretreated with or without NAC (5 mM) 2 h prior to treatment with CCM (40 *μ*M) for 48 h. (a) Vitality staining of spheroids (from left to right: phase-contrast image (i), fluorescent images of FDA(+) living cells in green (ii), PI(+) dead cells in red (iii), and merged (iv)). (b) The spheroid viability was measured by the enhanced cell viability assay kit. (c) The levels of apoptosis- and necroptosis-mediated proteins were analyzed by Western blotting. ^∗^*P* < 0.05 vs. respective control cells. ^#^*P* < 0.05 vs. respective CCM-treated cells. CCM: curcumin; NAC: N-acetylcysteine.

To ensure whether the results of 2D monolayer cultures were consistent in 3D cultures, spheroids derived from PC-3 and PC-3AcT cells were treated with 40 *μ*M curcumin for 48 h. Live and dead cells were stained with FDA and PI, respectively, and visualized under a Nikon Eclipse fluorescence microscope. Figures 6(a) and 6(b) show that curcumin treatment reduced spheroid growth, increased necrotic core, and decreased spheroid viability in both cell types. However, these events were blocked by NAC pretreatment. To characterize the nature of cell death, we investigated the effects of curcumin on the levels of apoptosis- and necroptosis-related proteins.  Figure 6(c) shows that curcumin treatment increased the levels of p-RIP3 and p-MLKL proteins as well as Bax and cleaved forms of caspase-3 and PARP proteins but decreased the level of Bcl-2. Curcumin-induced changes in these proteins were significantly recovered by NAC treatment.

## 4. Discussion

As part of an investigation into curcumin's anticancer potential, the response of PC-3 and PC-3AcT cells to curcumin when they were exposed to acidic stress was examined. Our results showed that curcumin produced preferential cytotoxic effects on PC-3AcT cells preadapted with lactic acid, as evidenced by cell viability assay (Figure 1(a)), measurement of ROS (Figure 1(b)) and mitochondrial membrane potential (Figure 1(c)), and subG_0_/G_1_ peak in the cell cycle (Figure 2(a)), and annexin V-PE binding assay (Figure 2(c)). The concurrent induction of apoptosis and necroptosis of PC-3AcT cells in an acidic environment with curcumin treatment was also demonstrated in a 3D spheroid cell culture model.

*In vitro* cytotoxicity results showed that acid-resistant PC-3AcT cells exhibited high levels of resistance to docetaxel and increased sensitivity to curcumin compared to parental PC-3 cells. This supports the results of earlier studies that extracellular acidification may promote resistance to chemotherapeutic drugs [3, 5]. Tumor cells are known to be able to uptake lactate and oxidize as a fuel source under low glucose conditions [20]; thus, in cultures that contain enough glucose, lactic acid appears to mainly contribute to extracellular acidification rather than entering the cells for energy supply. Cancer cells of lactic acidosis have been found to shift glucose metabolism from aerobic glycolysis (Warburg phenotype) to oxidative phosphorylation (Warburg phenotype) [21]. Therefore, the mechanism of action of curcumin that induces mitochondrial dysfunction by targeting mitochondria may serve as a basis for explaining preferential cytotoxicity by curcumin to PC-3AcT cells. In this process, excessive ROS production and ATP loss due to mitochondrial dysfunction seem to be important factors in determining cell death in PC-3 and PC-3AcT cells treated with docetaxel or curcumin.

Chemotherapy drugs used in PC, including cabazitaxel, can cause DNA damage and produce ROS [22], which can cause collateral damage to healthy noncancerous cells. In this regard, the concentration of curcumin used in this study showed preferential cytotoxicity to PC-3 and PC-3AcT cells with little damage to the two normal prostate epithelial cell lines RWPE-1 and HPrEC tested. High levels of ROS are detrimental to cells. Normal cells do not easily reach the death threshold due to the low levels of basal ROS and high antioxidant capacity, whereas cancer cells with elevated ROS levels can effectively reach the death threshold by any compounds or drugs that alter the redox balance and increase oxidative stress [23]. These findings are supported, at least in part, by our data that curcumin led to preferential cytotoxicity for acid-tolerant PC-3AcT cells, which indicates a further increase of ROS levels.

Oxidative stress is triggered by an imbalance between ROS production and removal by antioxidants, which causes oxidative damage to several cellular structures, such as DNA, proteins, lipids, and membranes. Among the organelles, mitochondria are a major site of ROS generation and are vulnerable to ROS damage [24]. In the present study, curcumin was effective in increasing ROS levels and inducing significant loss of *ΔΨ*m, indicative of mitochondrial dysfunction, in PC cells, which was prevented by pretreatment with NAC. Impairment of energy metabolism due to mitochondrial dysfunction eventually inhibits the production of cellular ATP. Compared to PC-3 cells, a further decrease in ATP levels in curcumin-treated PC-3AcT cells reflects the extent of mitochondria damage, indicating that it eventually resulted in an increase in cell death. Mitochondria are the essential organelles for maintaining cellular energy balance and regulating cell survival and death. Loss of *ΔΨ*m reduces the coupling efficiency of electron transport chain, thereby decreasing cellular ATP levels and contributing to cell death [25]. Therefore, the induction of apoptosis and necroptosis after curcumin treatment seems to be associated with a decrease in cellular ATP levels due to mitochondrial dysfunction. ATP supplemented externally in cancer cells is known to be internalized through macropinocytosis and other endocytic processes to promote cancer growth, survival, and drug resistance [26]. In our study, recovery of cell viability along with reduction of apoptosis- and necroptosis-related markers by ATP supplementation supports previous evidence that cellular ATP levels control the fate of apoptosis or necroptosis [25].

Induction of apoptosis through ROS may explain some of the anticancer mechanism(s) of curcumin *in vitro*, in various cancers [27, 28]. Excessive ROS production is known to cause single- or double-strand breaks and trigger DNA damage responses by activating ATM-CHK2 or ATR-CHK1 signaling pathways [29]. The activation of cell cycle checkpoints and repair mechanisms in response to DNA damage results in cell cycle arrest, but in the case of severe damage, it promotes apoptosis following cell cycle arrest. Our results demonstrated that curcumin led to DNA damage, as evidenced by the increased phosphorylation of ATM(Ser^1981^), ATR(Ser^428^), CHK1(Ser^345^), and p-CHK2(Thr^68^), as well as histone H2A.X(Ser^139^) as a marker for double-strand breaks. Thus, the delay in cell cycle transition at the G_2_/M phase, along with the presence of DNA damage response and apoptosis, present in this study, suggests DNA damage that exceeds DNA repair capacity in curcumin-treated PC-3 cells.

Oxidative stress induces necroptosis [30]. Curcumin has been reported to have a cytoprotective effect by attenuating necroptosis in some normal cells, including neurons [31], but the induction of necroptosis for cancer cells that was observed in the present study has not been previously described. The relevance of necroptosis in curcumin-induced cytotoxicity was further established by the increased levels of necroptosis markers, including p-RIP3 and p-MLKL. Inhibition of necroptosis by pharmacological inhibitor necrostatin-1 effectively blocked curcumin-mediated cell death along with the reduction of necroptotic markers in PC cells. Further, the addition of NAC reversed these changes by curcumin at the cellular and molecular levels. In spheroid cultures treated with curcumin, increased PI-positive regions were observed, indicating apoptotic and necrotic cell death, which was recovered by NAC pretreatment. In particular, the results of 3D spheroid viability assay and changes in cell death markers in spheroids indicate that the nature of cell death induced by curcumin was associated with concomitant apoptosis and necroptosis. Because 3D spheroids mimic solid tumors, they represent a useful *in vitro* model for confirming the results obtained in monolayer cell cultures [19]. Therefore, these data indicate that excessive ROS play key and regulatory roles in curcumin-mediated programmed cell death such as apoptosis and necroptosis following DNA damage and mitochondrial dysfunction. Further studies are required to characterize possible cross-talk between apoptotic pathway and necroptosis.

## 5. Conclusion

Curcumin exerted significant cytotoxic effects on PC-3 cells by inducing ROS production, DNA damage and consequent mitochondrial dysfunction, and ultimately apoptosis and necroptosis. While the underlying mechanisms of necroptosis in cancer treatment need to be comprehensively investigated, our data may improve the understanding of the novel role of curcumin for prostate carcinoma cells growing, especially in an acidic microenvironment.

## Data Availability

The data used to support the findings of this study are available from the corresponding author upon request.
